# Why Do We Study Aquatic Organisms?

**DOI:** 10.3390/ijms242115807

**Published:** 2023-10-31

**Authors:** Malgorzata Kloc, Jacek Z. Kubiak

**Affiliations:** 1 The Houston Methodist Research Institute, Transplant Immunology, Houston, TX 77030, USA; 2 The Houston Methodist Hospital, Department of Surgery, Houston, TX 77030, USA; 3 MD Anderson Cancer Center, Department of Genetics, The University of Texas, Houston, TX 77030, USA; 4 Laboratory of Molecular Oncology and Innovative Therapies, Military Institute of Medicine—National Research Institute (WIM-PIB), Szaserow 128, 04-141 Warsaw, Poland; 5 Dynamics and Mechanics of Epithelia Group, Institute of Genetics and Development of Rennes, Faculty of Medicine, University of Rennes, CNRS, UMR 6290, 35043 Rennes, France

Aquatic organisms comprising various plant and animal taxa represent a wide range of adaptations to a specific environment, but they also share many features with nonaquatic organisms of a given taxonomic group. These properties make them an ideal model system for scientific studies in various areas of medicine, evolution, adaptation, development, pharmacology, toxicology, and environmental biology. Below are a few examples of the most current studies on aquatic organisms with potential biological and medical applications.

Recent studies in the frog *Xenopus tropicalis* showed that the expression of peroxidase genes (Prdx) which encode a family of antioxidant proteins have a very distinct temporal pattern during early development [1]. Peroxidases are heme-containing glycoproteins that utilize either H_(2)_O_(2)_ or O_(2)_ to oxidize various substrates and can protect cells from oxidative damage. These enzymes are also commonly used in various diagnostic assays and industrial enzymatic reactions. Peroxidase gene promoters are also used to breed beneficial plants, and Prdx gene overexpression can confer tolerance to salt or oxidative stress [2]. A study in another frog species, *Xenopus laevis*, identified a new gene, Crb3.L, which belongs to the Crumbs (Crbs) family of transmembrane proteins which recruit actin linkers and promote apical membrane growth. Using the multiciliated cells (MCCs) of a *Xenopus* embryo epidermis which, in coordination with an actin cytoskeleton, reshape their apical domain to grow cilia, authors showed that Crb3 is necessary for the centriole migration and organization of the apical domains of multiciliated cells. Multiciliated cells (MCCs) occur, for example, in the spinal cord and the ventricles of the human brain, where they drive the flow of cerebrospinal fluid. In airways, MCCs are involved in the clearance of mucus. In the oviduct, MCCs participate in the transport of eggs. Thus, MCC dysfunction causes diseases of the brain, airway, and reproductive tracts [3]. Another aquatic model, the zebrafish *Danio rerio*, was recently used to uncover the role of insulin signaling in development [4] and the genetic and epigenetic regulation of retinal Müller glial cells, which are the main glial cells in retinae [5]. A zebrafish model of fatty liver disease was recently used to study the role of lipid genes in liver disease [6]. These findings in the frog and fish models have major implications for human health and diseases.

Humans can also benefit from the study of aquatic organisms via the medicinal contributions of marine organisms such as sponges, ascidians, corals, algae, bacteria, and fungi. These organisms are sources of novel anti-HIV, anti-bacterial, anti-trypanosomal, and anti-plasmodial medications [7]. Aquatic organisms are also the hosts of many unique symbionts, which are invaluable sources of metabolites with bioactive properties and can be mined for drug discovery and medicinal chemistry. Recently, seven novel compounds were isolated from the *Spongia officinalis*-derived fungal endosymbiont *Penicillium verruculosum*. One of them, averufin, demonstrated very strong anticancer activity against myeloid leukemia [8]. In addition to being a source of medically applicable compounds, some aquatic organisms are important food sources, supporting the livelihoods of coastal communities worldwide. Sea cucumbers represent one such example. These soft-bodied echinoderms have great nutritional, pharmaceutical, and medicinal value. They are rich in proteins, essential amino acids, vitamins, vitamins, minerals, collagen, omega-3, minerals, collagen, and polyunsaturated fatty acids but low in fat, cholesterol, and sugar. They are also used to produce tonic medicines, such as the gamat oil used to treat minor wounds and muscle and joint pain [9,10]. The overharvesting of marine organisms has dire consequences for the environment by reducing its capacity to recycle nutrients and diminishing ocean acidification, lowering symbiont biodiversity, and affecting organic matter recycling. To minimize overharvesting and its effects, recent studies on sea urchins postulated using discarded sea cucumber viscera as an agricultural feed additive [10].

Sea cucumbers and other Echinoderms are excellent models for studying organ and tissue regeneration and adult cell renewal. Echinoderms have the remarkable ability to regenerate most of their adult tissues, including the central nervous system [11,12]. This also makes them a potential source of regenerative and wound-healing compounds for clinical applications [13,14,15]. The regeneration capability is not limited to invertebrates but also occurs in aquatic vertebrates such as the axolotl, Ambystoma mexicanum [16,17,18]. The regeneration response is also used as a model for highly sensitive ecotoxicological tests for exposure to endocrine disrupters and as toxicological sentinels for the marine ecosystem [19,20]. The endocrine-disrupting compounds derived from pharmaceuticals, cosmetics, and packaging, such as parabens, pesticides, triclosan, polychlorinated biphenyls, bisphenol A, and phthalates, mimic the functions of hormones. They are abundant in water systems and have a detrimental effect on animal and human organs and systems, including the thyroid and reproductive system, causing thyroid hyperactivity, premature menopause and ovarian failure, endometriosis, uterine fibroids, polycystic ovary syndrome, and infertility [21,22]. Since estrogenic endocrine disruptors cause the feminization of *Xenopus* males [23] and craniofacial abnormalities in zebrafish [24], these aquatic species are also very useful ecotoxicology models. 

These are just a few examples that rationalize why the study of aquatic organisms is important for human health and well-being and the environment. We hope that further studies on various aspects of the biology, development, physiology, biochemistry, and genetics of aquatic organisms will not only enhance basic knowledge but will also be sources of novel anti-microbial and anti-cancer agents and bioactive compounds for wound healing and tissue and organ regeneration. They will also help in the assessment of the effects of water pollutants on human health.
